# Dynamics of Insulin Signaling in the Black-Legged Tick, *Ixodes scapularis*

**DOI:** 10.3389/fendo.2019.00292

**Published:** 2019-05-21

**Authors:** Arvind Sharma, Rana Pooraiiouby, Blanca Guzman, Preston Vu, Monika Gulia-Nuss, Andrew B. Nuss

**Affiliations:** ^1^Department of Biochemistry and Molecular Biology, University of Nevada, Reno, NV, United States; ^2^Department of Agriculture, Veterinary, and Rangeland Sciences, University of Nevada, Reno, NV, United States

**Keywords:** insulin, tick, ILPs, peptide, blood-feeding

## Abstract

Insulin-like peptides (ILPs) have been identified in several invertebrates, particularly insects, and work on these ILPs has revealed many roles including regulation of energy homeostasis, growth, development, and lifespan to name a few. However, information on arthropod ILPs outside of insects is sparse. Studies of Ixodid tick ILPs are particularly scarce, despite their importance as vectors of infectious agents, most notably Lyme disease. The recent publication of the genome of the black-legged tick, *Ixodes scapularis*, has advanced opportunities to study this organism from a molecular standpoint, a resource sorely needed for an organism with challenging life history requirements for study in the laboratory, such as a long life cycle and obligate, prolonged, blood-feeding at each life stage. Through bioinformatics searches of the tick genome and other available *I. scapularis* databases, we identified four putative ILP sequences. Full-length sequences of these ILP transcripts were confirmed, and quantitative RT-PCR was used to examine expression levels of these ILPs in different life stages, feeding states, and adult tissues. This work serves as an initial characterization of ILP expression in ticks and provides the foundation for further exploration of the roles of ILPs in these important arthropod vectors.

## Introduction

Insulin-like peptides (ILPs) are recognized to have several important functions in arthropods. This has been most extensively characterized in the dipterans *Drosophila melanogaster* and *Aedes aegypti* (1–4). Insects have multiple ILPs, ranging from as few as four in planthoppers (5), to eight in *Ae. aegypti* and *D. melanogaster* (2, 6, 7), to over 30 in *Bombyx mori* (8). The functions of these ILPs in most arthropods are largely unexplored, but, where investigated, ILPs have demonstrated pleiotropic roles, regulating metabolism, growth, reproduction, longevity, and larval molt, among other functions (9–11). Tissue expression of ILPs is also variable, with many produced in nervous tissue for neurohemal release. Others are produced in the midgut, fat body, or imaginal discs (7, 12, 13).

ILPs are variable in amino acid sequence, but are united in sharing conserved cysteine residues consistently spaced throughout the molecule. These cysteines form disulfide bridges, folding the peptide into a domain structure required for insulin receptor (IR) activation. The synthesis and post-translational processing of ILPs also is conserved. A prepropeptide is translated with B, C, and A chains (14). After disulfide bonds form, the C-chain is processed at dibasic cleavage sites, leaving behind the joined B and A chains. Some speculate that the C-chain is left intact in certain arthropod ILPs as is the case with human Insulin-like Growth Factor I (IGF-I) (2). However, to date, few native arthropod ILPs have been isolated and none of these examples retain the C-peptide (15).

Rapid genome and transcriptome sequencing, and associated bioinformatics processing of such data, have opened up previously unavailable resources for the study of neuropeptides and peptide hormones in arthropods. Our initial understanding of insect ILP structure, function, and signaling dynamics is the result of the isolation of bombyxin from *B. mori* (16) and *Locusta* Insulin-Related Peptide (LIRP) from *Locusta migratoria* (17). Characterization of individual ILPs through painstaking genetic methods such as PCR amplification with degenerate primers eventually gave way to available genomic and transcriptome databases. The availability of these sequences has permitted the use of genetic techniques such as RNAi and quantitative PCR to advance our knowledge of specific ILP functions. However, extraction, quantification, and synthesis of the ILP molecules themselves for study has remained a challenge for many arthropods.

The above mentioned research on ILPs is dominated by more easily studied model insects, not to mention the exhaustive vertebrate literature on insulin, IGFs, and relaxin, and little information on neuropeptide signaling in arachnids exists. In ticks, immunocytochemical evidence for the presence of ILPs has been reported in *Ornithodoros parkerii* (18) and *Rhipicephalus appendiculatus* (19) using an antibody to bombyxin. Immunostaining in *Dermacentor variabilis* using an anti-insulin antibody demonstrated a decrease in immunoreactivity in the synganglion after blood-feeding and mating (20). A partial fragment of an apparent A-chain of a *D. variabilis* ILP was detected by 454 pyrosequencing of the transcriptome (21), and a subsequent study showed a decrease in expression of this ILP in partially fed and replete ticks in comparison to unfed ticks (22). In *Amblyomma americanum*, upregulation of an ILP was detected in pathogen-challenged ticks (23), and knockdown of Insulin-like Growth Factor Binding Protein (IGFBP) in this tick prevented blood-feeding females from feeding to repletion (24).

In the United States, ticks are responsible for 95% of vector borne diseases (25). The publication of the genome sequence of *Ixodes scapularis*, the black-legged tick, has provided a wealth of information on this important vector (26). This was a much needed resource in our efforts to prevent the debilitating diseases transmitted by these ticks and others, including Lyme disease, babesisosis, anaplasmosis, ehrlichiosis, Rocky Mountain Spotted Fever, Southern Tick-Associated Rash Illness, Tick-Borne Relapsing Fever, tularemia, Colorado tick fever, Powassan encephalitis, and Q fever (25). In addition to its utility as a way to study vector-pathogen interactions, the *I. scapularis* genome and transcriptomes provide a unique opportunity to examine neuropeptides in a non-model arthropod. Therefore, in this study, we characterized the sequence and expression dynamics of ILPs in *I. scapularis*, to provide a basis for further explorations of these signaling molecules in ticks.

## Methods

### Bioinformatics Identification of *Ixodes scapularis* ILPs

Amino acid sequences from previously identified ILPs from *Ae. aegypti, Anopheles stephensi, An. gambiae, D. melanogaster*, and vertebrate ILPs [*Mus musculus* insulin and insulin-like growth factor I (IGF-I)] were used as queries in tBLASTn searches of the *I. scapularis* genome and expressed sequence tag (EST) databases, and *I. ricinus* transcriptome (TSA) databases through the National Center for Biotechnology Information (NCBI) website. To ensure detection of ILPs that might deviate from canonical insect ILP sequences, these queries were further searched against all available arthropods, from whole genome sequencing reads, transcriptomes, and EST databases. From these results, putative ILP amino acid sequences from representative arthropods outside of Hexapods were selected with an emphasis on arachnids (e.g., Aranae, Acari, Scorpiones, Opilionies, Amblypygi, Pseudoscorpiones, Solfugae), but also including the outgroup Merostomata (Atlantic Horseshoe Crab, *Limulus polyphemus*) (Supplementary Table 1). These were then leveraged as queries in searches against the *I. scapularis* genome and EST database to identify ILP signatures potentially unique to non-Hexapod arthropods.

Analyses of signal peptide sequences within putative prepropeptides were conducted using both the Neural networks and the Hidden Markov models contained in the SignalP program [http://www.cbs.dtu.dk/services/SignalP/ (27)].

### *I. scapularis* Colony

A colony of *I. scapularis* was maintained at 20°C under 16:8 light:dark conditions at 95% humidity. Larvae and nymphs were fed on mice as described previously (28). Adults were fed on rabbit (29). All animals were treated humanely according to the guidelines of the Institutional Animal Care and Use Committee (IACUC protocol # 00682).

### Sample Collection and RNA Extraction

RNA was extracted from three separate biological cohorts of *I. scapularis* eggs (~500), unfed larvae (10 individuals), nymphs (10 individuals), adult female (2 individuals), and adult male (5 individuals) ticks by placing them separately into 1.7 ml tubes, and extracting with TRIzol™ reagent (Invitrogen), following a slightly modified protocol from the manufacturer's instructions. The modified protocol is as follows: *I. scapularis* were collected into 1.7 ml tubes and were ground in liquid nitrogen with autoclaved plastic pestles. These samples were further homogenized in 100 μl of TRIzol™ with a tissumizer, then brought to 800 μl with additional TRIzol™. Solid tissue debris was pelleted by centrifugation at 12,000 x g at 4°C for 10 min, and the supernatant was transferred to a fresh tube. Thereafter, the manufacturer's protocol was followed for extraction of total RNA.

The resulting RNA pellet was resuspended in 20 μl of RNase/DNase free water incubated at 55–60°C for 10 min. RNA was treated with DNase I from the Direct-zol^TM^ RNA MiniPrep Kit (Zymo Research) at RT for 30 min. Columns from this kit were pre-wet with RNA Wash Buffer, briefly centrifuged, then the DNase-treated RNA was added directly to the columns. Manufacturer protocols were followed for subsequent RNA recovery. Total RNA quantity was measured with Nanodrop spectrophotometer. Purity was determined by 260/280 and 260/230 ratios, with acceptable values in the ~1.8 and 2.0–2.2 range, respectively (all samples collected met purity standards). RNA samples were immediately stored at −80°C.

### Sequencing Confirmation of *I. scapularis* ILPs

To confirm sequences, primers were designed to the identified *I. scapularis* ILP sequences using Primer3 (30, 31) and estimated optimal annealing temperatures were determined using Integrated DNA Technologies' online Oligoanalyzer tool (IDT) (Table 1). Synthesis of *I. scapularis* cDNA was accomplished using the SMARTer cDNA synthesis kit according to manufacturer's instructions (Takara Bio USA, Mountain View, CA) and 1 μg total RNA obtained from nymphs. These cDNA template and primers were used in polymerase chain reactions (PCR) to amplify sections of *I. scapularis* cDNA using Biotool Taq polymerase (Biotool, Ely, UK) with the following program: 40 cycles at 94°C, 30 s; 60°C, 30 s; 72°C, 1 min. This included an initial 5 min denaturing step at 94°C, and a final 10 min, 72°C extension step. These reactions were separated with gel electrophoresis and bands of the expected size were extracted from the gel and sent for Sanger sequencing (Genewiz, South Plainfield, NJ). Resulting sequences were aligned with the predicted sequences.

**Table 1:** Primer sequences used to amplify IsILP sequences for confirmation by Sanger sequencing or for relative quantification by qRT-PCR.

**Primer**	**Nucleotide sequence**
**SEQUENCING PRIMERS**	
NotI d(T)2	AACTGGAAGAATTCGCGGCCGCAGGAATTTTTTTTTTTTTTTTTTTTTTTTTTTTTTVN
NotI-d(T)	AACTGGAAGAATTCGCGGCCGCAGGAATTTTTTTTTTTTTTTTTT
ILP1F2	GATCGGTCAGCGACTGTTTC
ILP1Fwd5	TTTCCTGGGCACTCAACAC
ILP1R210	GGACACCAGAGGAAACAGTC
ILP3F3	TGCTCGACTTCCTCTGTGAAG
ILP3R3	CAGTACGCGAGAAGCTCCAG
ILP4F2	CTCTAGCCTGGACGCCTTC
ILP4Fwd87	AACCACCGAAGAACAACAGC
ILP4R1	GAGGGCCTGTAGATCACGAA
ILP5F1	CGGAGATGACGGACCTGTTC
ILP5Fwd343	AGCTCGTCTCACACATGAAC
**RT-QPCR PRIMERS**	
ILP1Fwd131	AGGCAGAGTTCTACGATCCG
ILP1Rev210	GGACACCAGAGGAAACAGTC
ILP3Fwd108	CTTTTGAGGCTCTGCCAAGA
ILP3Rev201	CCCGTGCGCTTGTTGTTATC
ILP4Fwd87	AACCACCGAAGAACAACAGC
ILP4Rev189	GCCTCCACCAGGTCTACGAA
ILP5Fwd343	AGCTCGTCTCACACATGAAC
ILP5Rev452	CAGGAACAGGTCCGTCATCT
β-tubulinFwd	TGAATGACCTGGTGTCCGAG
β-tubulinRev	GACAAGCTGTTCAAGCCTCT

To confirm 5′ and 3′ ends of ILP transcripts not accessible to primer design, 5′ and 3′ rapid amplification of cDNA ends (RACE) was performed. For 3′ RACE, the anchor primer NotI d(T)2 (Table 1) was used to initiate cDNA synthesis using SMARTer cDNA synthesis kit and 1 μg total RNA from blood-fed nymphs. This template was used in nested PCR reactions as above, using gene-specific reverse primers and the NotI anchor primer. For 5′ RACE, specific ILP reverse primers were used with the SMARTer cDNA synthesis kit for generation of cDNA. These cDNA strands were purified and the 3′ end was polyadenylated (A) using Terminal Deoxynucleotidyl Transferase (TdT) and dATP (Thermo Fisher Scientific Inc.). Poly-A tailed cDNA was again isolated and used as template in nested PCR reactions employing NotI d(T)2, NotI and gene specific reverse primers. Resulting products were separated by gel electrophoresis and subcloned using the TOPO™ TA Cloning™ Kit for Subcloning (Invitrogen, Waltham, MA, USA). Plasmids were isolated from successful transformants and were Sanger sequenced as above.

### Expression of *I. scapularis* ILPs

ILP expression was characterized using quantitative RT-PCR (qRT-PCR). The iScript^TM^ cDNA Synthesis Kit was used to convert 500 ng−1 μg *I. scapularis* total RNA samples from eggs, larvae, nymphs, and male and female adults (see above) into cDNA following manufacturer's protocols (BioRad, Hercules, CA, USA). Briefly, cDNA of ticks from different life stages was diluted 5x with deionized H_2_O before using it as a template in qRT-PCR experiments. One microliter cDNA was used in each 10 μl qRT-PCR reaction. Four different housekeeping genes were tested: β-tubulin, ubiquitin, ribosomal protein 4 (rps4), and I13A. β-tubulin was selected for final experiments because of its consistent expression in all life stages and tissues. Sequences of specific primers for IsILPs and the housekeeping gene, β-tubulin, are listed in Table 1. Each sample was run in triplicate wells of 96-well plate. qRT-PCR was performed on CFX touch Real-Time PCR Detection system using SYBR green master mix (BioRad, Hercules, CA, USA). All reactions were performed with initial 5 min at 95°C, followed by 40 cycles of 10 s at 95°C, 15 s at 64°C, and 15 s at 72°C, and a melt curve was analyzed at 70–95°C. Relative expression was calculated using 2^−ΔΔ*Ct*^ method. Experiments were replicated two to four times with different tick cohorts.

### Statistical Analysis

All data were analyzed using GraphPad Prism 7 software (La Jolla, CA, USA). Relative expression values of IsILP expression in different developmental stages and tissues were compared with one-way ANOVA, followed by Dunnett's multiple comparison test.

## Results

### *I. scapularis* ILP Identification

Four sequences with ILP characteristics were identified through bioinformatics searches of *I. scapularis* genome, transcriptome, and EST databases. The use of mosquito ILPs as queries in tBLASTn searches was sufficient to detect these sequences and no additional sequences were found with subsequent searches employing other arthropod ILPs. Sequences containing complete features of ILPs (start and stop codons, B and A chains, and signal peptide) were found for three *I. scapularis* ILPs. For IsILP4, only a partial sequence was obtained for *I. scapularis* which contained no stop codon. However, a highly similar transcriptome sequence from *I. ricinus* (accession: GANP01013821) was found that did contain a stop codon and untranslated regions further downstream. Reverse primers designed to these regions in the *I. ricinus* sequence were capable of amplifying PCR products in combination with forward primers from *I. scapularis*.

**Figure 1:**
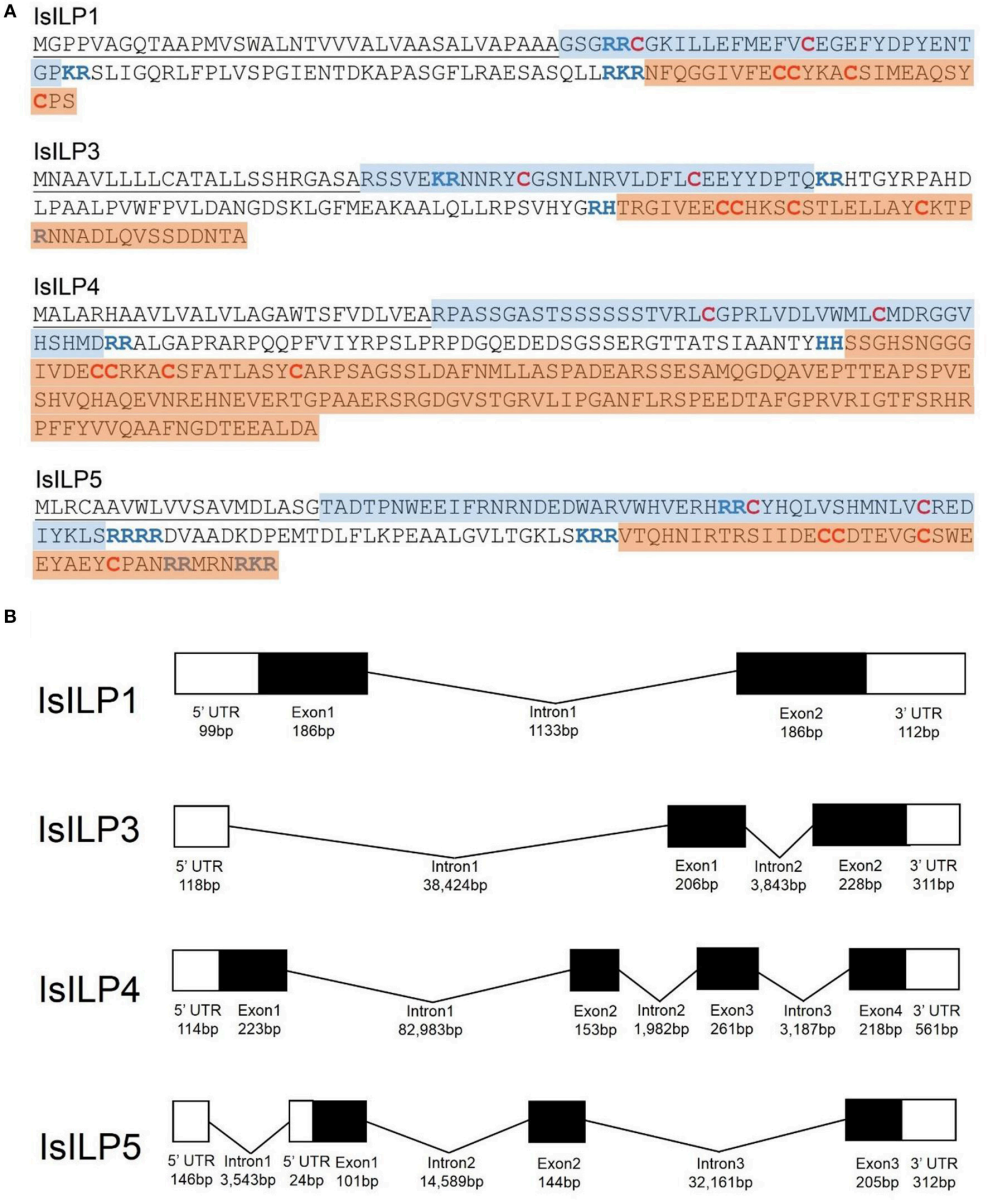
Structural features of *I. scapularis* ILPs. **(A)** Amino acid translations and structural predictions of *I. scapularis* ILP sequences confirmed by Sanger sequencing. Cysteine disulfide bridges in bold red text. Predicted di-basic cleavage sites in bold blue text; B-chain: Blue highlight; A-chain: Orange highlight; Signal peptide underlined. Genbank accession numbers: IsILP1: MK649814; IsILP3: MK649815; IsILP4: MK649816; IsILP5: MK649817; **(B)** Predicted gene models of IsILPs based on alignment of confirmed sequences to the *I. scapularis* genome (IsILP1, IsILP3: Contig: PKSA02008623, IsILP4: Contig: PKSA02013804, IsILP5: Contig: PKSA02002782). Intron and exon lengths in the diagram are not proportional.

**Figure 2:**
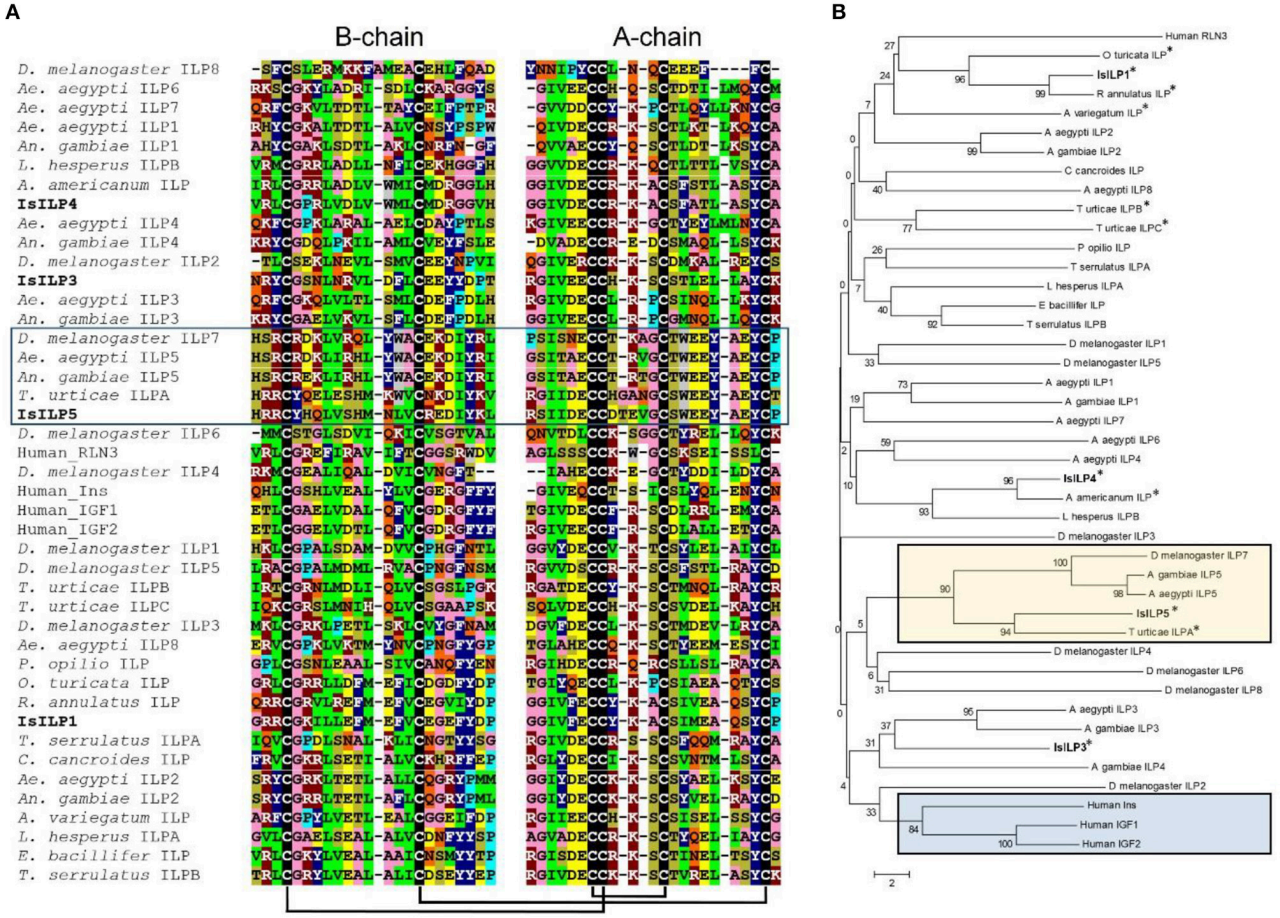
Comparison of IsILP amino acid sequences to human ILPs and selected arthropods. **(A)** Alignment of *I. scapularis* B- and A-chain ILP amino acid sequences (truncated around core Cys resides for the sake of space) with selected arachnid, insect, and human ILPs. Colors indicate amino acids with similar properties: green = hydrophobic; dark blue = aromatic; pink = small; dark red = basic; yellow = acidic, tan = hydroxyl, gray = tryptophan, light blue = proline, black = cysteine, orange = amide. Predicted disulfide bridges are connected by black bars. Box: ILP5 sub-group. Alignments performed in Clustal Omega (32) and colored manually. **(B)** Neighbor-joining tree of mosquito ILP A and B chain amino acid sequences comparing selected arachnid, insect, and human ILPs. Acari ILPs indicated by “^*^”. Tan box: ILP5-related sequences; Blue box: Human ILPs. Tree was constructed using MEGA version 6 (33). Accession numbers of sequences used for this figure can be found in Supplementary Table 2.

Complete cDNA sequences of four ILPs were confirmed by Sanger sequencing of PCR products and 3′ and 5′ RACE products. These sequences contain features consistent with ILPs in other animals, including start and stop codons, signal peptides, conserved cysteine residues, and putative dibasic cleavage sites (Figures 1, 2). IsILP1 was named as such considering it was the first *I. scapularis* ILP noted in the literature (21). The remaining ILPs (3, 4, and 5) were named according to their amino acid similarity to *An. gambiae* ILPs, particularly ILP5 which contains additional amino acids between key Cys residues of the A-chain in comparison to other ILPs (Figure 2 and see section Discussion).

We encountered but were unable to amplify a putative additional *I. scapularis* ILP sequence that contained spacing of cysteines characteristic of an ILP A-chain: ILVFFSVKPDKKPAFSSK**CC**DGD**C**TKSVWKGSKISFHVFLPAPFVRVRE (accession: ABJB010579748). This may represent a pseudogene or an unrelated sequence that was picked up in our bioinformatics search by chance, or genome assembly in this region is of insufficient quality to design functional primers.

### *I. scapularis* ILP Developmental Expression

**Figure 3:**
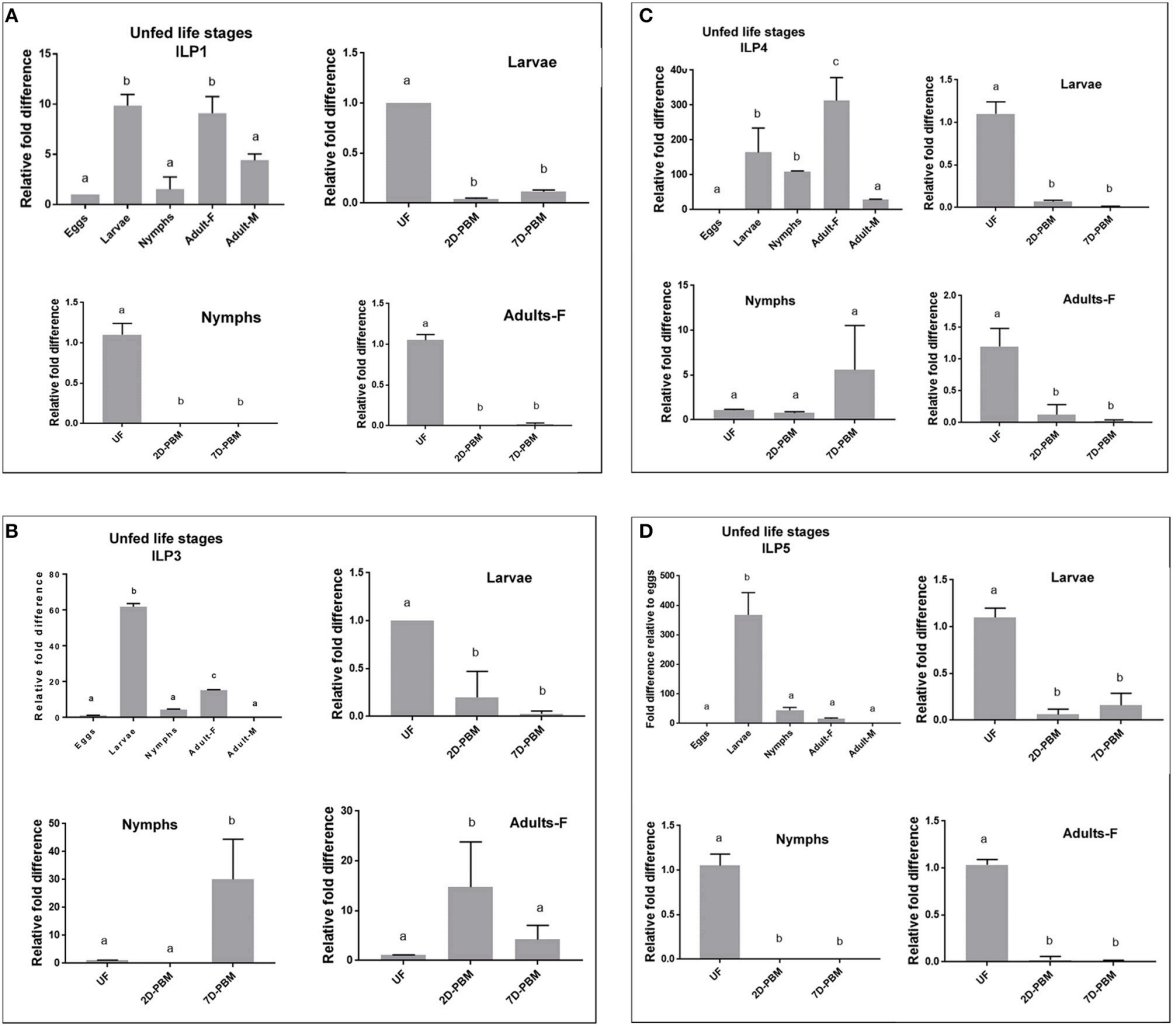
Expression of IsILP transcripts in *I. scapularis* life stages, as determined by quantitative RT-PCR. F, female; M, male; UF, unfed; 2D-PBM, 2 days post engorgement and host detachment; 7D-PBM, 7 days post engorgement and host detachment. Fold expression levels comparing life stages were scaled to expression in eggs. Feeding trials were examined within life stages by scaling expression to the unfed state. Treatments were examined by one-way ANOVA followed by Dunnett's multiple comparison test. **(A)** Expression of IsILP1 in unfed developmental stages and larvae, nymphs, and adult females post detachment. All unfed stages, *F*_(4,5)_ = 29.27, *P* = 0.001; Larvae: *F*_(2,3)_ = 4583, *P* < 0.0001; Nymphs: *F*_(2,3)_ = 121, *P* = 0.001; Adults: *F*_(2,3)_ = 410.2, *P* = 0.0002. **(B)** Expression of IsILP3 in unfed developmental stages and larvae, nymphs, and adult females post detachment. All unfed stages, *F*_(4,5)_ = 1968, *P* < 0.0001; Larvae: *F*_(2,3)_ = 21.46, *P* = 0.01; Nymphs: *F*_(2,9)_ = 16.9, *P* = 0.0001; Adults: *F*_(2,9)_ = 6.774, *P* = 0.01. **(C)** Expression of IsILP4 in unfed developmental stages and larvae, nymphs, and adult females post detachment. All unfed stages, *F*_(4,5)_ = 16.5, *P* = 0.004; Larvae: *F*_(2,3)_ = 111, *P* < 0.001; Nymphs: *F*_(2,8)_ = 3.002, *P* = 0.10; Adults: *F*_(2,3)_ = 24.25, *P* = 0.01. **(D)** Expression of IsILP5 in unfed developmental stages and larvae, nymphs, and adult females post detachment. All unfed stages, *F*_(4,15)_ = 101.9, *P* < 0.0001; Larvae: *F*_(2,6)_ = 99.08, *P* < 0.0001; Nymphs: *F*_(2,9)_ = 264.6, *P* < 0.0001; Adults: *F*_(2,6)_ = 661.4, *P* < 0.0001.

ILP expression varied markedly between the developmental stages sampled. For fold difference calculations, eggs samples were used as a baseline (control). IsILP1 was significantly more expressed in the unfed larvae and adult females compared to eggs, nymphs, and adult males. In contrast, IsILP3 and IsILP5 expression was significantly higher in unfed larvae as compared to other life stages. IsILP4 was significantly more expressed in adult females, followed by larvae and nymphs having significantly more expression than adult males and eggs. For most IsILPs, there was little to no expression in adult males and eggs (Figures 3A–D).

IsILP1 and IsILP5 expression was significantly higher in unfed stages (larvae, nymphs, and adults) compared to the levels at 2 and 7 days after detachment from the host (Figures 3A,D). For IsILP3, expression was highest in unfed larvae compared to detachment from the host (Figure 3B); whereas in nymphs and adult females expression increased after detachment from the host and was higher at 2-day post detachment in adults and 7 day post detachment in the nymphs. For IsILP4, expression was significantly higher in unfed larvae and adults, whereas there was no difference in expression before and after a blood meal in the nymphal stage (Figure 3C).

### *I. scapularis* ILP Adult Female Tissue Expression

Synganglion, salivary gland, midgut, and ovaries from unfed females were dissected to understand baseline tissue expression profiles of IsILPs. IsILP1 and IsILP5 had significantly higher expression in synganglion. IsILP5 expression was limited to synganglion. IsILP3 and IsILP4 expression was significantly higher in salivary glands. However, both expressed in all tissues examined.

**Figure 4:**
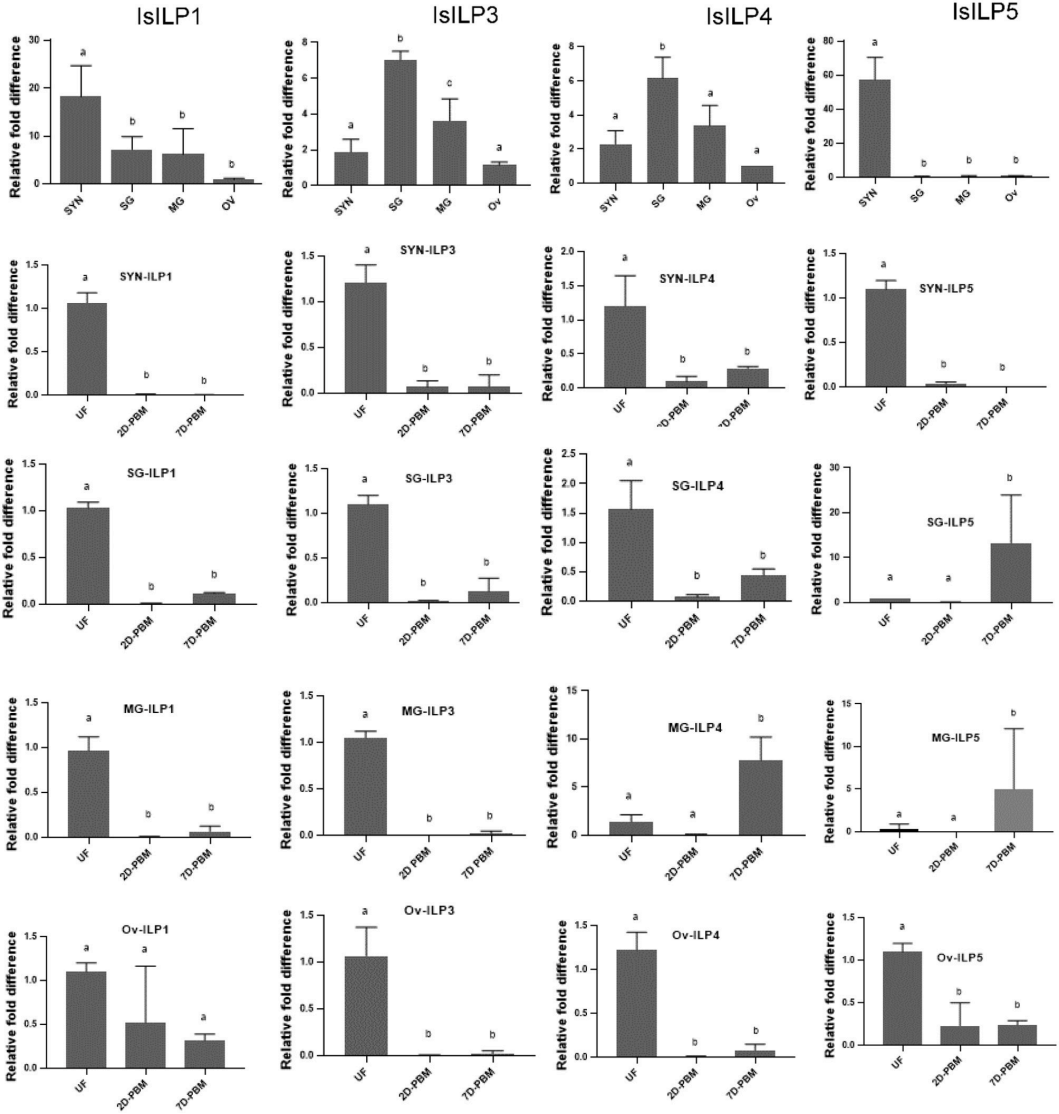
Expression of IsILP transcripts in adult female *I. scapularis* tissues, as determined by quantitative RT-PCR. SYN, synganglion; SG, salivary gland; MG, midgut; Ov, ovaries; UF, unfed; 2D-PBM, 2 days post engorgement and host detachment; 7D-PBM, 7 days post engorgement and host detachment. Top row: Expression of individual IsILPs in unfed adult females compared by body tissue. Fold expression levels were scaled to expression in ovaries and were examined by one-way ANOVA followed by Dunnett's multiple comparison test. IsILP1 unfed tissues, *F*_(3,8)_ = 8.156, *P* = 0.008; IsILP1-Synganglion: *F*_(2,6)_ = 252.1, *P* < 0.0001; IsILP1-salivary glands: *F*_(2,6)_ = 836.8, *P* < 0.0001; IsILP1-midgut: *F*_(2,6)_ = 97.47, *P* < 0.0001; IsILp1-Ovaries: *F*_(2,6)_ = 3.491, *P* = 0.09. IsILP3 unfed tissues, *F*_(3,7)_ = 25.42, *P* = 0.0004; IsILP3-Synganglion: *F*_(2,6)_ = 68.23, *P* < 0.0001; IsILP3-salivary glands: *F*_(2,6)_ = 98.0.8, *P* < 0.0001; IsILP3-midgut: *F*_(2,6)_ = 374.6, *P* = 0.0003; IsILP3-ovaries: *F*_(2,6)_ = 35.26, *P* = 0.0005. IsILP4 unfed tissues, *F*_(3,7)_ = 14.26, *P* = 0.002; IsILP4-Synganglion: *F*_(2,6)_ = 15.17, *P* = 0.004; IsILP4-salivary glands: *F*_(2,6)_ = 21.64, *P* = 0.001, IsILP4-midgut: *F*_(2,5)_ = 28.4, *P* = 0.001; IsILP4-ovaries: *F*_(2,6)_ = 94.22, *P* < 0.0001. IsILP5 unfed tissues, *F*_(3,8)_ = 57.74, *P* < 0.0001; IsILP5-Synganglion: *F*_(2,6)_ = 340.3, *P* < 0.0001; IsILP5-salivary glands: *F*_(2,7)_ = 5.176, *P* = 0.04, IsILP5-midgut: *F*_(2,5)_ = 1.73, *P* = 0.2; IsILP5-ovaries: *F*_(2,6)_ = 26.32, *P* < 0.001.

IsILP1 expression was reduced after a blood meal in all tissues except for ovaries where no difference was noted in expression before or after feeding. IsILP3 expression was highest in unfed females in all tissues examined whereas IsILP4 expression was higher in midgut 7 days post detachment from the host. IsILP5 expression was highest in the synganglion of the unfed females. We noted some expression in ovaries; however, the Ct values were too high to accurately measure expression (36–38 out of 40 cycles) suggesting very little transcript was available. IsILP5 expression was higher in a few midgut and salivary gland samples at 7 day post detachment (Figure 4).

## Discussion

To date, little examination has been made of ILPs in arachnids and our characterization of ILPs in *I. scapularis* provides a foundation for further exploration of the roles of these neuropeptides in this medically important arthropod. The discovery of four ILPs is consistent with the pattern seen in insects of multiple ILPs. Some of the *I. scapularis* ILP sequences we characterized have been previously noted as ILPs in the literature or as automated annotations in online databases. For instance, putative transcripts for IsILP1 were noted from *I. scapularis* (34) that were highly similar to an A-chain fragment from *D. variabalis* (21). However, this is the first study to specifically focus on IsILPs, and IsILPs 3 and 4 are identified for the first time in our study. It is of interest that only IsILP5 (ISCW002549, partial fragment annotated as ILP4), and not IsILP1, was initially annotated in the *I. scapularis* genome (26). Also, an ILP sequence was recently recognized from *A. americanum* that is highly similar to IsILP4, yet this did not map to the *I. scapularis* genome (23). This highlights the large and scattered nature of the current *I. scapularis* genome and the challenge of assembling and annotating a genome with extensive repetitive sequences and expansive intron regions.

The IsILPs we identified fit the characteristic hallmarks of ILPs in other organisms, containing signal peptides, B and A chains, and dibasic cleavage sites for excision of the C-peptide. Conserved cysteine residues are predicted to form disulfide bridges between B and A chains or internally in the A chain (Figures 1A, 2A). Insulin-like growth factors (IGFs) have a similar structure, yet retain a short C-peptide that is not cleaved (11). None of the IsILPs in this study appear to have a short C-peptide indicative of an IGF-like structure. Some other commonalities for ILPs include basic (Arg, Lys, and His) and amino acids with aromatic rings (Tyr and His) upstream of the first Cys of the B-chain, and consistently spaced hydrophobic (particularly Leu) or charged residues (frequently Glu, but also Asp). IsILP5, AgILP5, AaILP5, and DmILP7 are unusual in that they have additional amino acids between Cys 2 and 3 of the A-chain compared to other ILPs, where there are consistently three amino acids (Figure 2A). These particular ILPs share considerable amino acid sequence conservation in comparison to other ILPs, particularly in the A-chain. Outside of this particular sub-group, IsILPs vary greatly in their similarity to each other and to those of other arthropods (Figure 2). As has been the case with other invertebrate ILPs, amino acid sequence similarity alone provides only limited information as to their potential functions when comparing between species. It is likely that the secondary structure imposed by disulfide bonds and as yet unverified key amino acid residues may preserve the functionality of these molecules in binding to their cognate IRs. Unfortunately, the chemical synthesis of invertebrate ILPs has been a challenge and has so far limited the study of structure-activity relationships with techniques such as alanine screens (3, 15).

The IsILPs we identified in this study had from two to four exons (Figure 1B), comparable to other arthropod examples (*A. gambiae*: 1–3 exons; *Ae. aegypti*: 2–3 exons; *D. melanogaster*: 1–3 exons) (35, 36). Curiously, the ILP with the most conserved amino acid structure among the arthropods we examined, ILP5 (DmILP7), exhibited high variability in intron number between organisms (AgILP5: 0 introns; AaILP5: 1 intron; DmILP7: 2 introns; IsILP5: 3 introns). The gene structure of IsILP1 was consistent with previous predictions in *I. scapularis* of a single intron (21). IsILPs 3, 4, and 5 are predicted to have massive introns, ranging from 32,161 to 82,983 bp. However, this is not inconsistent with observed intron lengths in other organisms such as IGF1 in humans, which has a 55,952 bp intron (35). Both IsILP1 and IsILP3 were found on the same contig (PKSA02008623), but curiously the entire IsILP1 gene sequence was found within intron 1 of the IsILP3 sequence. We encountered another contig containing both IsILP1 and IsILP3 (PKSA02013879) with a similar gene structure, but with different intron lengths for IsILP3 (Intron 1: 35,843 bp; Intron 2: 3,773 bp). It is unclear whether the current genome assembly accurately reflects the gene structure of these IsILPs. This confusion may be clarified with newer sequencing technologies permitting longer reads allowing more robust genome assemblies.

Perhaps of greater value toward understanding IsILP functions are the expression patterns of ILPs. In specific body tissues, IsILP5 was almost exclusively expressed in the synganglion suggesting a neurotransmitter or neurohemal role. IsILP1 was also expressed at significantly higher levels in the synganglion compared to other tissues. In contrast, IsILPs 3 and 4 were expressed in all tissues examined with highest expression in the salivary glands. Tick salivary glands undergo a growth phase during feeding in order to increase saliva production and then greatly reduce in size after host detachment. As some ILPs act as growth factors, one might speculate higher expression in these tissues may indicate synthesis and storage of these ILPs in preparation for initiation of a feeding event, where their release subsequently stimulates salivary gland growth.

Expression of most ILPs decreased after a blood meal which is similar to a previous study where expression of an ILP (the homolog of IsILP1) dropped during and after a blood meal in *D. variabilis* females (22). In our study, this occurred in most tissues and in most life stages. The importance of a reduction in IsILP transcripts in response to feeding is, as yet, unclear, but may be tied to regulation of energy storage as is the case for ILPs in other organisms. Also of interest are exceptions to this trend, such as the increase in expression of IsILP4 and IsILP5 in the midgut of adult females 7 days post blood meal. The increase in expression in this tissue after a blood meal may suggest a role of IsILPs 4 and 5 in blood digestion, a situation similar to that seen in *Ae. aegypti* where AaILP3 secreted from the brain stimulates the midgut to produce the digestive enzyme trypsin following a blood meal (4). In addition, nymphs increased expression of IsILPs 3 and 4 after detaching from hosts, possibly suggesting a developmental role unique to this life stage. A greater number of timepoints over a longer period may further elucidate the role of IsILPs before, during, and after blood feeding, and during development.

In adult *I. scapularis* males, IsILP expression was low for all ILPs except IsILP1. The primary activity for adult males of this species is to survive long enough to find and mate with a female. While this activity is most successful if the males cling to and search a vertebrate host where a prospective female is likely to be attached for feeding, the males themselves do not feed. Considering the major functions of ILPs identified in other animals include storage of nutrients, endocrine cascades resulting in yolk protein synthesis, and growth, the lack of IsILP expression in males may make sense in that they do not engage in these processes in the adult stage.

In addition to ILP expression levels in ticks, binding proteins, which were not investigated in this study, may also play a role in insulin signaling dynamics. For instance, knockdown of IGFBP in *A. americanum* prevented blood-feeding females from feeding to repletion (24). Although they are not ILPs, IGFBPs do interact with ILPs and can regulate binding dynamics with the insulin receptor by altering degradation rates and binding kinetics. Exploration of the expression dynamics of IGFBPs and their impact on IsILP stability in *I. scapularis* will provide further information on the roles of these signaling molecules in tick physiology. ILPs also have been implicated in immune responses, and expression of an ILP (homolog of IsILP1) was upregulated in *A. americanum* exposed to *Ehrlichia chaffeensis*, the causative agent of human monocytic ehrlichiosis (23). Whether similar expression patterns occur in *I. scapularis* in response to pathogens remains to be explored, but it is a subject of particular interest given the role of insulin signaling in immunity in other blood-feeding arthropods (37), and the importance of *I. scapularis* as a vector of disease.

## Conclusions

Ticks represent an extreme lifestyle compared to many animals. They feed exclusively on vertebrate blood, and Ixodid ticks feed only three times in their entire life cycle. From these scant meals, they can endure many months between feedings, yet still manage to deal with the harsh realities of the environment as other arthropods do, but without the luxury of regular food intake. No doubt absorption and conservation of energy is critical to their survival and ILPs are likely to play a key role in this process. We are eager to uncover the role of these signaling molecules with regard to the extreme physiology of these arachnids, and this study provides an important first step in the characterization and role of these neuropeptides.

## Author Contributions

AN and MG-N wrote the draft manuscript, conceived the experiments, and wrote the final manuscript. RP and PV amplified and sequenced the ILP transcripts. AS and BG carried out the insulin transcript expression study. MG-N performed the statistical analysis.

### Conflict of Interest Statement

The authors declare that the research was conducted in the absence of any commercial or financial relationships that could be construed as a potential conflict of interest.
