# Supplementary material for: Dynamics of Insulin Signaling in the Black-Legged Tick, Ixodes scapularis
Source: Front Endocrinol (Lausanne). 2019 May 21;10:292. doi: 10.3389/fendo.2019.00292 (PMC6536706; doi:10.3389/fendo.2019.00292)
Supplement: Supplementary file 1 [file Table_1.docx]

Supplemental Table 1: Selected arthropod ILP sequences (fragments or complete sequences) identified via tBLASTn searches of the NCBI database with a focus on arachnid ILP sequences. Sequences from *I. scapularis* are noted in red with corresponding ILP nomenclature from this study.

| Class | Order | Family | Species | Accession # |
| --- | --- | --- | --- | --- |
| Arachnida | Scorpiones | Buthidae | *Isometrus maculatus* | EU252378 |
|  |  |  | *Centruroides exilicauda* | AXZI01120476 AXZI01196488 AXZI01196487 AXZI01180220 |
|  |  |  | *Mesobuthus martensii* | AYEL01069806 |
|  |  |  | *Tityus serrulatus* | GBZU01012395.1  GBZU01008129.1 |
|  | Araneae | Theridiidae | *Latrodectus hesperus* | GBJN01163744.1 GBJN01143433.1 |
|  |  | Eresoidea | *Stegodyphus mimosarum* | GAZR01025972 GAZR01004432 |
|  |  | Lycosidae | *Pardosa pseudoannulata* | GCKE01037689.1 GCVS01019250.1 |
|  |  | Sicariidae | *Loxosceles reclusa* | JJRW010362599.1 |
|  |  | Theraphosidae | *Acanthoscurria geniculata* | AZMS0104802491.1 GAZS01041613 |
|  | Opiliones | Phalangiidae | *Phalangium opilio* | GDAO01000770.1 |
|  | Solfugae | Daesiidae | *Gluvia dorsalis* | GDAP01001472.1 GDAP01007548.1 |
|  | Amblypygi | Phrynichidae | *Euphrynichus bacillifer* | GDAJ01000444.1 |
|  | Pseudoscorpionida | Cheliferidae | *Chelifer cancroides* | GDAK01003762.1 |
|  | Acari | Argasidae | *Ornithodoros turicata* | GDIC01001879.1 |
|  |  | Phytoseiidae | *Metaseiulus occidentalis* | XM_003743122 |
|  |  | Ixodidae | *Amblyomma variegatum* | BK007652 |
|  |  |  | *Dermacentor variabilis* | EU616823 |
|  |  |  | *Rhipicephalus annulatus* | GBJT01013022.1 |
|  |  |  | *Ixodes scapularis* | XM_002402930: IsILP1  GBBN01011597: IsILP3 GBBN01019325: IsILP4  EL515756: IsILP5 |
|  |  |  | *Ixodes ricinus* | GCJO01021265.1 GANP01013821 |
|  |  | Tetranychidae | *Tetranychus urticae* | GW057456 GW063394 JR696562 |
|  |  |  | *Panonychus ulmi* | GCAC01006825 |
|  |  | Achipteriidae | *Achipteria coleoptrata* | LBFM01021513 |
|  |  | Hypochthoniidae | *Hypochthonius rufulus* | LBFL01049295 |
|  |  | Camisiidae | *Platynothrus peltifer* | LBFO01006606 |
|  |  | Sarcoptidae | *Sarcoptes scabiei* | JXLN01013560 |
|  |  | Pyroglyphidae | *Dermatophagoides farinae* | ASGP01001954 |
| Merostomata | Xiphosura | Limulidae | *Limulus polyphemus* | FN226896 XM_013916704 XM_013929606 XM_013919035 XM_013916808 AZTN01177337 |
