# Supplementary material for: Dynamics of Insulin Signaling in the Black-Legged Tick, Ixodes scapularis
Source: Front Endocrinol (Lausanne). 2019 May 21;10:292. doi: 10.3389/fendo.2019.00292 (PMC6536706; doi:10.3389/fendo.2019.00292)
Supplement: Supplementary file 2 [file Table_2.docx]

Supplemental Table 2: Selected arthropod ILP sequences and accession numbers for Fig. 2. Organisms with multiple ILPs where these have not previously been described are designated with “A”, “B”, “C”, etc. rather than numbers.

| Species | Order | ILP name | Accession |
| --- | --- | --- | --- |
| *Aedes aegypti* | Diptera | AaILP1 | DQ845750 |
|  |  | AaILP2 | DQ845752 |
|  |  | AaILP3 | DQ845751 |
|  |  | AaILP4 | DQ845753 |
|  |  | AaILP5 | DQ845758 |
|  |  | AaILP6 | DQ845755 |
|  |  | AaILP7 | DQ845757 |
|  |  | AaILP8 | DQ845754 |
| *Amblyomma variegatum* | Acari |  | BK007652 |
| *Amblyomma americanum* | Acari |  | c56947_g1_i1 |
| *Anopheles gambiae* | Diptera | AgILP1 | AY324307 |
|  |  | AgILP2 | AY324308 |
|  |  | AgILP3 | AY324309 |
|  |  | AgILP4 | AY324310 |
|  |  | AgILP5 | AY324311–12 |
| *Chelifer cancroides* | Pseudoscorpionida |  | GDAK01003762.1 |
| *Drosophila melanogaster* | Diptera | DmILP1 | NM_140102 |
|  |  | DmILP2 | NM_079288 |
|  |  | DmILP3 | HE654173 |
|  |  | DmILP4 | HE654173 |
|  |  | DmILP5 | NM_206315 |
|  |  | DmILP6 | NM_130644 |
|  |  | DmILP7 | NM_130714 |
|  |  | DmILP8 | NM_140692 |
| *Euphrynichus bacillifer* | Amblypygi |  | GDAJ01000444.1 |
| *Homo sapiens* | Primates | Insulin | NP_000198 |
|  |  | IGF-I | P05019-1 |
|  |  | IGF-2 | P01344-1 |
|  |  | Relaxin-3 | NC_000019 |
| *Latrodectus hesperus* | Araneae | LhILPA | GBJN01163744.1 |
|  |  | LhILPB | GBJN01143433.1 |
| *Ornithodoros turicata* | Acari |  | GDIC01001879.1 |
| *Phalangium opilio* | Opiliones |  | GDAO01000770.1 |
| *Rhipicephalus annulatus* | Acari |  | GBJT01013022.1 |
| *Tetranychus urticae* | Acari | TuILPA | GW057456 |
|  |  | TuILPB | JR696562 |
|  |  | TuILPC | GW063394 |
| *Tityus serrulatus* | Scorpiones | TsILPA | GBZU01012395.1 |
|  |  | TsILPB | GBZU01008129.1 |
